# Lateral reading and monetary incentives to spot disinformation about science

**DOI:** 10.1038/s41598-022-09168-y

**Published:** 2022-04-05

**Authors:** Folco Panizza, Piero Ronzani, Carlo Martini, Simone Mattavelli, Tiffany Morisseau, Matteo Motterlini

**Affiliations:** 1 Molecular Mind Laboratory, IMT School for Advanced Studies Lucca, Lucca , Italy; 2 Centre for Applied and Experimental Epistemology, Vita-Salute San Raffaele University, Cesano Maderno, Italy; 3 TINT – Centre for Philosophy of Social Science, Department of Political and Economic Studies, University of Helsinki, Helsinki, Finland; 4 Department of Psychology, Bicocca University, Milano, Italy; 5 Université de Paris and Université Gustave Eiffel, LaPEA, Boulogne-Billancourt, France; 6 Strane Innovation, Gif-sur-Yvette, France

**Keywords:** Human behaviour, Psychology and behaviour

## Abstract

Disinformation about science can impose enormous economic and public health burdens. A recently proposed strategy to help online users recognise false content is to follow the techniques of professional fact checkers, such as looking for information on other websites (lateral reading) and looking beyond the first results suggested by search engines (click restraint). In two preregistered online experiments (N = 5387), we simulated a social media environment and tested two interventions, one in the form of a pop-up meant to advise participants to follow such techniques, the other based on monetary incentives. We measured participants’ ability to identify whether information was scientifically valid or invalid. Analysis of participants’ search style reveals that both monetary incentives and pop-up increased the use of fact-checking strategies. Monetary incentives were overall effective in increasing accuracy, whereas the pop-up worked when the source of information was unknown. Pop-up and incentives, when used together, produced a cumulative effect on accuracy. We suggest that monetary incentives enhance content relevance, and could be combined with fact-checking techniques to counteract disinformation.

## Introduction

Scientific disinformation is the intentional spreading of misleading or outright false content purporting to have a basis in scientific methods and practices. Circulation of inaccurate scientific information can damage both institutions and individuals, further affecting the relation of trust between science and society^1^. Successful misconceptions influence the public debate on decisions regarding the effectiveness of a vaccine, the adoption of solutions mitigating climate change, or the cost of a social policy. A prime example of the detrimental effects of scientific disinformation comes from the use of ivermectin, an oral drug that has been widely used in several countries as a treatment against COVID-19 disease, despite no evidence of clinical efficacy^2^. The sharing of false information is easily fuelled by political or social motivations that disregard the best scientific evidence on the matter.

There are structural challenges to fighting the spread of false or misleading information on social media. One key issue is that companies often perceive a trade-off between engaging users and monitoring viral but potentially fake content, to the point of favouring the former over the latter^3^. Contrasting disinformation is made even more difficult when there is a deliberate intent behind the dissemination. For example, at the peak of the coronavirus infodemic, only 16% of fact-checked disinformation was labelled as such by Facebook’s algorithms, partly because content creators were able to simply repost content with minor changes, thus escaping detection^4^. It is therefore essential that, in combination with a systematic change in policy, users themselves are empowered against malicious or false content. Lay evaluation of science-related disinformation is harder than other forms of disinformation (e.g. political) because in the former case the lines between expertise and pseudoexpertise are blurred, and incompetent or otherwise biased sources pose as expert sources on topics like epidemiology or climate change^5^.

Research on countering disinformation has developed substantially over the last decade, bringing a wealth of different approaches^6–10^. These include debunking, the systematic correction of false claims after they have been seen or heard^11,12^, pre-bunking, preventive measures before exposure to disinformation^7,13^, nudging, interventions affecting users’ choices without limiting their freedom of choice^14^, and boosting, the empowering of users by fostering existing competences or instilling new ones^14^. All of the above approaches have proven to be useful in a social media context, not least by adopting ingenious and innovative adaptations of classical paradigms. Debunking has been extensively studied, with several experiments focusing on the source^15–18^ and the timing^19^ of fact checking. Research has also explored whether evaluations of the quality of contents and sources can be delegated to the so-called wisdom of crowds, with encouraging results^20–23^ (for a less optimistic perspective, see^24,25^). Studies on pre-bunking have largely focused on the concept of inoculation^7,26^, namely exposing users to disinformation strategies in order to ease their recognition in future settings. Inoculation has demonstrated pronounced and lasting effects when introduced through games^27–30^. Nudging was also tested by showing warning labels for unchecked or false claims^31–34^, but also by priming users to pay attention to the accuracy of content they might be willing to share^35–37^ (however see^38^ for a critique of this approach). Finally, boosting was tested by presenting users with a list of news/media literacy tips or guidelines on how to evaluate information on-line^39–43^, producing some remarkable results and some non-significant ones.

A promising example of media literacy intervention has been carried out by researchers interested in understanding how fact checkers navigate information when evaluating unfamiliar sources^44^. Researchers catalogued fact checkers’ strategies and distilled them into an educational curriculum called Civic Online Reasoning^45,46^. In particular, fact checkers adopt two core strategies to avoid being biased in their search. The first strategy is lateral reading, namely leaving a website and opening new tabs along a horizontal axis to use the resources of the Internet to learn more about a site and its claims. Appearance of websites can sometimes mislead about their reliability, hence reading laterally helps to identify potential issues such as undisclosed interests or false credentials. The second core strategy is click restraint, that is, to sift through search results of a browser search before clicking on any link. Given the well-documented tendency to open the first results without looking further^47^, order of appearance is vulnerable to manipulation: websites can in fact improve their rank in the results to increase incoming traffic, a process called search engine optimisation. A restraint from clicking thus prompts users to explore the various sources to discern which ones are the most trustworthy. Lateral reading and click restraint seem particularly fit when a content has unknown origins that are hard to identify or that appear legitimate on the surface, a feature that has been associated with content creators spreading scientific disinformation^48^.

In the absence of expertise and content knowledge, users can rely on a number of external cues to infer whether information presented as scientific is reliable^49^. Lateral reading and click restraint can thus be used when scientific disinformation is deceptively sophisticated and difficult to detect. Indeed, training on Civic Online Reasoning has proven effective in countering disinformation among high school and college students^50–52^, as well as elderly citizens^53^. Despite extensive research on Civic Online Reasoning, so far little attention has been paid to the application of these techniques on social media. It is therefore unclear how effective presenting these strategies on a social network can actually be.

Critical thinking strategies might not be the only potentially effective tools in evaluating scientific (dis)information. For instance users might not be sufficiently motivated to evaluate the truthfulness of the content they see^6,54,55^. Many users might share news simply because they come from a source they trust or like, or because those news align with their values, without paying much attention to accuracy. The spread of scientific disinformation then is not only related to false beliefs, but also to motivated behavior, paired with strong personal identities and values. In order to better exploit the benefits of critical thinking tools, it is therefore also important to identify the respective effects of being aware of truth-motivated strategies; i.e., being motivated to know the truth about a given topic. It may be that people, while being somehow familiar with fact-checking techniques, are only eager to apply them when identifying the truthfulness of the information is reinforced by specific incentives.

One way to test the effect of motivation then is the use of monetary incentives. In other words, does paying participants for their being accurate increase their accuracy in the evaluation of content? The idea behind this intervention is that money increases motivation, and thus the attention paid to otherwise ignored cues about the accuracy of content. A 40-year meta-analysis^56^ points out that both monetary incentives and intrinsic motivation predict performance, and that incentives are particularly relevant when they are directly tied to performance. Moreover, a study conducted in a setting comparable to the present experiment showed that monetary incentives are the main driver for people to spend time solving online tasks even in the face of small average earnings^57^.

Monetary incentives have been proven to be a cost effective tool to modify behavior in domains such as health and human development^58^, where often an early boost in motivation promotes the adoption of cheap preventive behaviours, avoiding this way costly consequences^59^. From a psychological perspective, the use of incentives builds on the attention-based account of disinformation spread. This account posits that certain features of social networks favour the dissemination of interesting and unexpected content at the expense of accuracy^6,60^. Recent research in this field has found both laboratory and field evidence that accuracy of content is often overlooked and that simple cues reminding participants to evaluate the accuracy of content reduce participants’ willingness to share fake news^35,37,61–63^ (or possibly increase true news sharing^38^). Increasing accuracy through incentives is not an entirely novel idea in social media either, as shown in a recent initiative promoted by Twitter^64^. Although these premises indicate that this type of intervention can be effective, it is not a given that economic incentives will have a positive effect on scientific content evaluation. In an experimental setting in particular, social media content is subject to higher scrutiny than when users scroll through their news feed^35^. It is therefore possible that additional incentives may not further increase participants’ accuracy.

**Figure 1:**
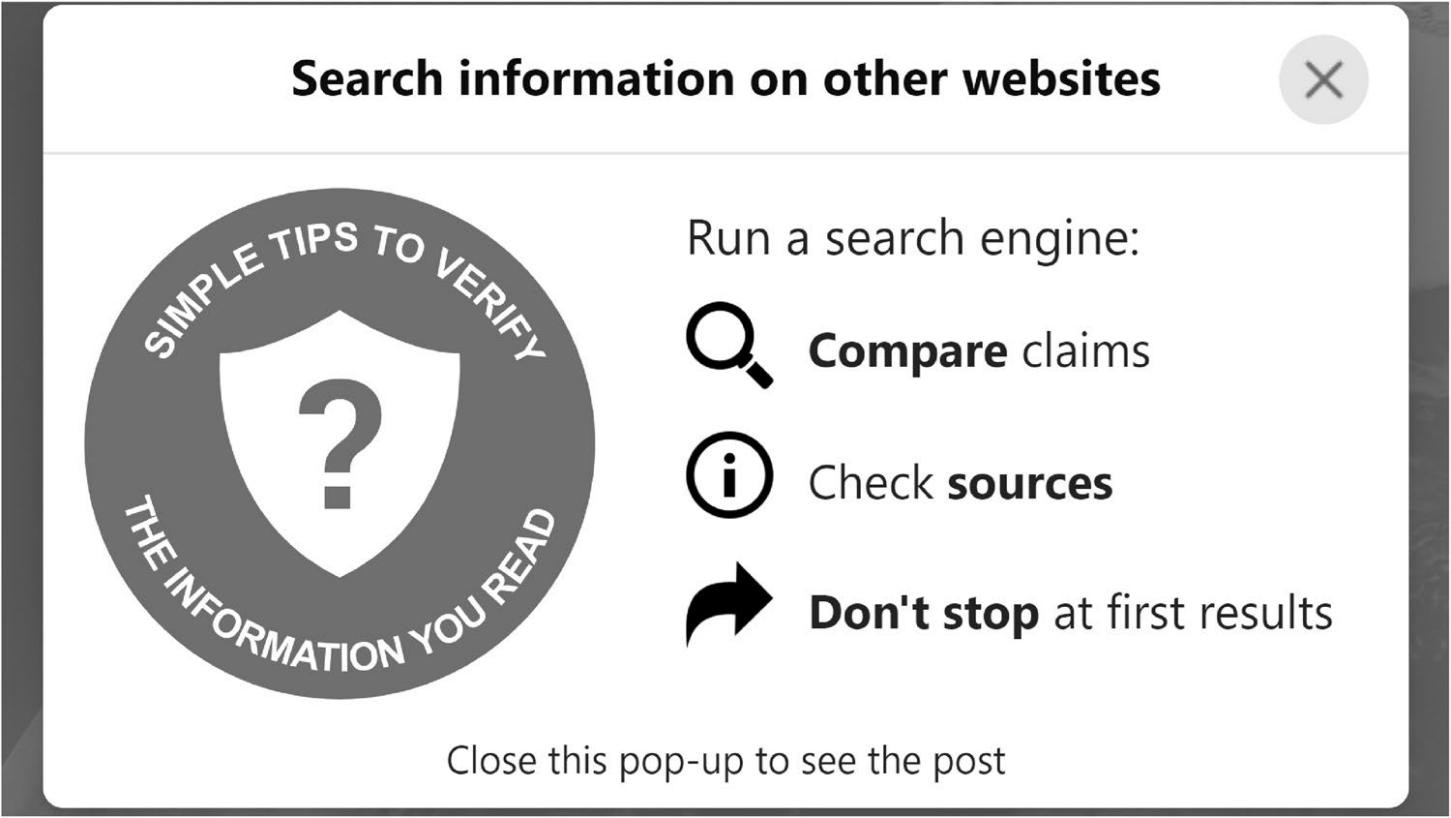
Screenshot of the pop-up presented to participants.

The aim of the present study was to test and compare the effectiveness of Civic Online Reasoning techniques and monetary incentives in contributing to the recognition of science-related content on social media. We conducted two pre-registered experiments where participants observed and interacted with one out of several Facebook posts that linked to an article presenting science-themed information. Participants were free to conduct further research on external websites in order to form a more accurate idea of the scientific validity of the post. Once satisfied with the information they gathered, participants rated how scientifically valid the claims contained in the post were. To test for the usefulness of Civic Online Reasoning techniques, we designed a pop-up that preceded the post presenting the lateral reading and click restraint strategies Fig. 1. The use of a pop-up ensured that participants processed the content before observing the post, an approach that has also been adopted in previous research^62^. A pop-up could be easily adapted in a social media setting as regular reminders with the necessary precautions to avoid the reduction of their salience with time^65,66^. To test the effect of monetary incentives instead, we doubled the participation fee (equivalent to an average +£8.40/hour) if participants guessed correctly the validity of the post they were evaluating.

## Experiment 1

In Experiment 1, we tested separately the efficacy of pop-up and monetary incentives, and compared their effects to a control condition with no interventions. To assess that the effect of the interventions is effective over the widest possible range of contexts, we used a set of 9 different Facebook posts varying in various properties, such as the scientific topic, the source reputation, and its level of factual reporting. The original pre-registration of this experiment can be retrieved from osf.io/gsu9j.

### Materials and methods

#### Ethics statement

All participants gave their written informed consent for participating in the experiment. The experimental protocols were approved by the Research Ethics Committee (CER) at the University of Paris (IRB No: 00012021-05), and all research was performed in accordance with the relevant guidelines and regulations.

#### Participants

We recruited 2700 U.K. residents through the online platform prolific.co on 11 March 2021 (for a rationale of sample size, see S1 Methods ). Average age was 36 ($SD=13.5$, 8 not specified), 60.7% of participants were female, (39.1% male, 0.2% other), and 55.6% had a Bachelor’s degree or higher. Although recruitment explicitly specified that the experiment was supported only on computers or laptops, 316 participants (11.7%) completed the experiment on a mobile device. As our hypotheses were based on the assumption that search would happen on a computer (where internet browsing easily allows to read laterally), both stimuli and measures were not designed for mobile use. We therefore had to exclude these participants from the analyses. Analyses were thus conducted on 2384 participants.

#### Design

**Figure 2:**
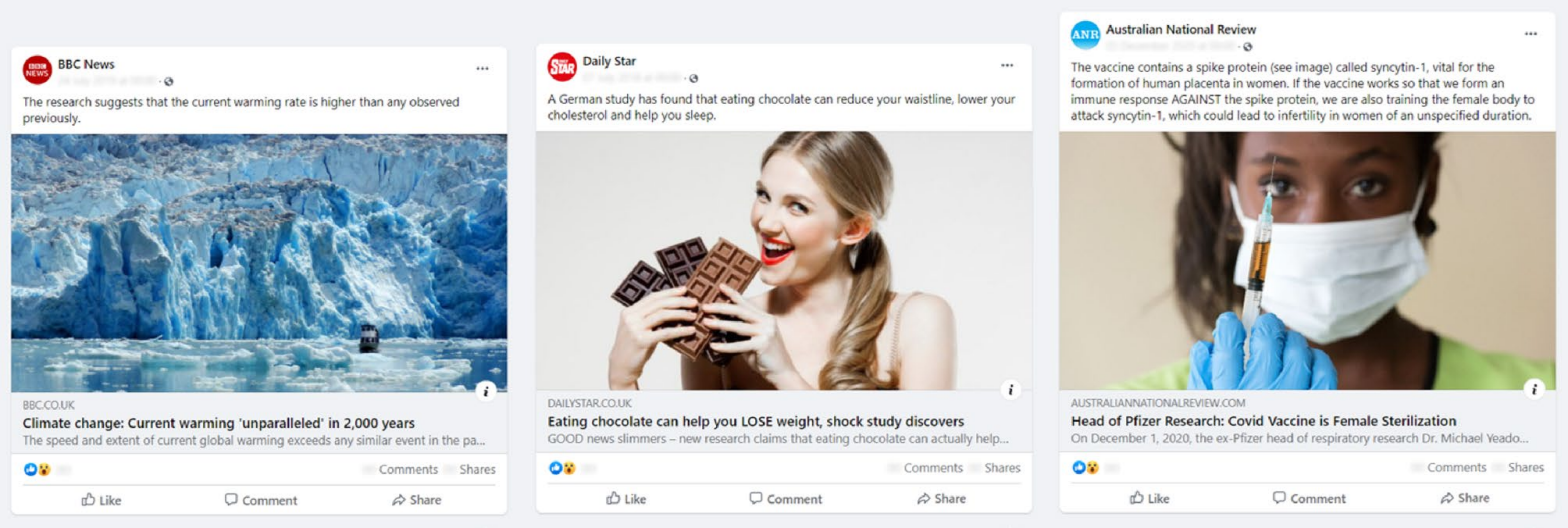
Examples of the stimuli presented, varying in topic (Climate Change, Health and Nutrition, COVID-19), factual reporting (high, low, low), scientific validity (high, low, low), and source reputation (trusted, untrusted, unknown source).

We conducted the experiment on Qualtrics and lab.js^67^. During the experiment, participants observed and were able to interact with one out of several Facebook-like posts (Fig. 2 shows three examples; click here for an interactive example from Experiment 2). Participants’ task was to rate the scientific validity of the statements reported in the title, subtitle, and caption of the post (“how scientifically valid would you rate the information contained in the post?”; 6-point likert scale from (1) “definitely invalid” to (6) “definitely valid”). Researchers rated independently the scientific validity of the posts’ content in terms of valid/invalid according to pre-specified criteria (see S3 Methods ). Participants could take as much time as they wanted in giving their rating. Crucially, participants were also explicitly told that they were allowed to leave the study page before evaluating the post. After the rating, participants completed a questionnaire and were paid £0.70 for their time. Median completion time of the experiment was 5 minutes.

*Experimental conditions* Participants were randomly assigned to one of three experimental conditions: control, incentive, and pop-up. In the control condition, participants completed the task as described above. In the incentive condition, participants were doubled their participation fee if their rating matched that given by the experimenters. Unknown to participants, the correctness of the answer depended only on whether the answer was valid or invalid, and not on the extremity of the answer (e.g. having answered 4 instead of 5), even though we selected unambiguously valid or invalid content. In the pop-up condition, presentation of the post was preceded by a pop-up (Fig. 1) presenting a list of civic online reasoning techniques (e.g., lateral reading, click restraint) as tips to verify the information in the post.

*Stimuli* Each participant observed one out of nine possible Facebook posts (Fig. 2; see S1 File for a full list). Posts varied in terms of: (i) scientific validity of the content (i.e., six valid and three invalid posts, either with verified or debunked information; S3 Methods ); (ii) topic (i.e., three on climate change, three on the coronavirus pandemic, three on health and nutrition); (iii) factual reporting of the source, based on ratings from mediabiasfactcheck.com (i.e., three high/very high versus six low/very low); (iv) source reputation, as measured in a screening survey (S4 Methods ; three categories: trusted (2 posts), distrusted (4), unknown source (3)). Posts were balanced to have three posts for each topic, one from a source with high factual reporting displaying valid information, one from a source with low factual reporting displaying valid information, and one from a source with low factual reporting displaying invalid information.

We standardised emoji reactions across all posts to control for their influence. In addition, post date, number of reactions and shares were blurred. The rest of the post was instead accessible to the participant, who could click on different links to access the source Facebook page, the original article, and the Wikipedia page (if present). Text and images were taken from the article and are publicly available (original links: osf.io/ces8g for Experiment 1 and osf.io/xsr43 for Experiment 2). Captions were short statements of a scientific nature, i.e. facts or events pertaining to some scientific mechanism.

#### Measures

*Accuracy* We computed two measures of accuracy–correct guessing and accuracy score. Correct guessing refers to a dichotomous variable that tracks whether participant gave a ’valid’ (vs. ’invalid’) rating when the post content was actually scientifically valid (vs. invalid). Accuracy score instead is a standardised measure ranging from zero to one, with 0 indicating an incorrect “1” or “6” validity rating, 0.2 indicating an incorrect “2” or “5” rating, 0.4 an incorrect “3” or “4” rating, 0.6 a correct “3” or “4” rating, 0.8 a correct “2” or “5” rating, and 1 a correct “1” or “6” rating. Accuracy score allows to distinguish validity evaluations that are associated with different behaviours: for instance, not all participants would be willing to share content that they rated as 4 in terms of scientific validity. In addition, accuracy score is statistically more powerful than correct guessing as it includes more possible responses^68^. We thus considered accuracy score as our main index.

*Search behaviour* During the evaluation of the post, we tracked participants’ behaviour on the study page. We measured the time spent both inside and outside the page, and a series of dummy variables tracking whether participants had clicked on any of the links present (e.g., Facebook page, article page, Wikipedia page). Based on these calculations we were able to estimate participants’ response times and search behaviour.

*Civic online reasoning* After having rated the scientific validity of the post, participants completed a questionnaire investigating those factors that could have influenced their choice. In order to test our hypotheses, we asked participants whether they engaged in lateral reading and click restraint. Participant were said to have used lateral reading if they reported having searched for information outside the study page (yes/no question), and if they specifically searched on a search engine among other destinations (multiple selection question). Participants were said to have used click restraint if they further reported looking beyond the first results suggested by the search engine (multiple choice question). Critically, questions were formulated in such a way as to avoid any expectation as to which answer to select, and thus reduce the influence of the experimenter.

*Control measures* In addition to measures of accuracy and civic online reasoning, we included a series of control measures for our analyses (S5 Methods ). Other questions included self-report measures of confidence in the validity rating, plausibility of the post content, subjective relevance of obtaining accurate information about the post, familiarity with the source, perceived trustworthiness of the source, subjective knowledge of the topic, trust in scientists, conspiratorial beliefs, and a scientific literacy test. In addition to responses in the questionnaire, we obtained information about participants from the recruiting platform, such as their level of education, socio-economic status, social media use, and belief in climate change.

#### Analyses

Statistical tests were conducted using base R^69^. We adopted the standard 5% significance level to test against the null hypotheses. All tests were two-tailed unless otherwise specified. Post-hoc tests and multiple comparisons were corrected using the Benjamini-Hochberg procedure, and 95% confidence intervals were also family-wise corrected. Non-parametric statistics were log-transformed for conciseness. For probability differences, the lower boundary indicates the 2.5% quantile of the effect of the target variable starting from the 2.5% quantile of the baseline probability estimate, whereas the upper boundary indicates the 97.5% quantile of the effect of the target variable starting from the 97.5% quantile of the baseline probability estimate. Given the small number of stimuli ($N<10$), we do not cluster errors by Facebook post in our regression analyses. The use of random effects yields however comparable results in magnitude and statistical significance unless otherwise reported.

#### Deviations from the pre-registered protocol

Although we tried to be as faithful as possible to the original pre-registered protocol, we made some changes which we report here:

- Scientific validity labels: labels for 1 and 6 responses were changed from “completely” to “definitely” invalid/valid
- Exclusion of mobile users: we anticipated that participants would have accessed the experiment exclusively through a computer or laptop, and we explicitly defined this as a requirement to participate in the study. Some participants however did participate using a mobile device. For this reason we had to introduce an additional exclusion criterion, use of a mobile device (see Participants).
- Effect of interventions on accuracy score: we report an ordinary logistic regression (Effect of interventions), originally listed as exploratory analysis, *in lieu* of the pre-registered ANOVA test. We deemed preferable to report a non-parametric test due to the strong violation of normality of the dependent variable. The ANOVA analysis yields the same results; it is reported in S1 Analyses.
- Effect of interventions on correct guessing: to test correct guessing, preregistered analyses proposed the use of a probit regression. We chose however to report results of a logistic regression for ease of comparison with the other tests reported, considering that the two regressions yielded the same results.

With the exception of the above-mentioned deviations, we conducted our analyses as described in the original pre-registration.

### Results

Participant randomisation was balanced across conditions (Chi squared test, $\chi ^2(2)=0.016$, $p=.99$). Median time to evaluate the Facebook post was 33 seconds in the control condition (incentive condition: 45 seconds; pop-up condition: 35 seconds; minimum overall time: 2 seconds, maximum overall time: 40 minutes). In the pop-up condition, participants spent an additional median time of 11 seconds on the pop-up. On a scale from 1 to 6 (3.5 response at chance level), average accuracy score in the control condition was 4.35 ($SD=1.20$; incentive condition 4.48, $SD=1.32$; pop-up condition 4.35, $SD=1.19$). In the control condition, 78.2% of participants correctly guessed the scientific validity of the post (incentive condition: 80.1%; pop-up condition: 78.1%).

#### Effect of interventions

To test the effect of our interventions on accuracy, we adopted two tests, one for the accuracy scores, and one for correct guessing (original preregistered analyses are presented in S1 Analyses ; se also Deviations from the pre-registered protocol). Since accuracy scores were clearly non-normally distributed (Shapiro-Wilk test, all $p<0.001$), we used an ordinal logistic regression in place of the linear regression to test the effect of condition on accuracy scores. Results showed a significant effect of incentive ($\beta =0.293$ [0.092, 0.494], $z=3.225$, $p=0.003$) and a lack of significance for the pop-up ($\beta =-0.009$ $[-0.207, 0.188]$, $z=-0.103$, $p=.918$). According to the model, the probability of giving a “definitely valid” (“definitely invalid”) correct response increases by 4.4% [1.5%,8.2%] in the incentive condition compared to the control condition. Exploratory analyses suggest that incentives were particularly effective in increasing accuracy scores in valid posts (against control: $\beta =0.3582$ [0.07329, 0.6431], $z=3.268$, $p=0.003$; against pop-up: $\beta =0.3713$ [0.0913, 0.6514], $z=3.447$, $p=0.003$; S5 Analyses ). These last results should be taken with caution however, as posts from trusted sources were all presenting valid content.

#### Technique adoption

**Figure 3:**
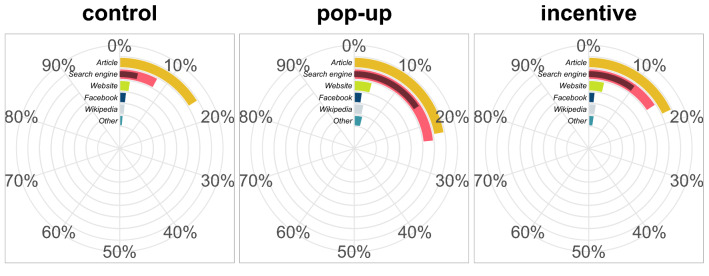
Race chart of self-report external search behaviour. Bars indicate the proportion of participants in each experimental condition reporting to have searched in either category of websites. Lateral reading is identified with the proportion of participants searching information on a search engine (light red), whereas click restraint is the subset of these participants who reported not stopping at the first algorithmically-ranked results of the search (dark red).

To compare the adoption of Civic Online Reasoning techniques between experimental conditions (pre-registered hypothesis 2) we used a logistic regression with technique use (adoption of both lateral reading and click restraint) as predicted variable and experimental condition as predictor. Results revealed that both incentive and pop-up increased technique adoption (Fig. 3; incentive: $\beta =1.042$ [0.527, 1.556], $z=4.728$, $p<0.001$; pop-up: $\beta =1.556$ [1.065, 2.046], $z=7.405$, $p<0.001$), but that the increase was markedly higher with the presence of the pop-up than with monetary incentives ($\beta =0.514$ [0.157, 0.871], $z=3.362$, $p<0.001$).

#### Exploratory: technique adoption

Since our measure of technique use is based on self-reporting, responses might have been biased by external expectations. We therefore checked whether participants who reported the use of techniques actually left the study by tracking their behaviour on the post’s web page. According to our measures, 80% of these participants left the study in the control condition, compared to 87% in the pop-up and 90% in the incentive conditions. This result, if anything, suggests that our interventions did not increase the rate of false reporting. Moreover, even after accounting for false reports, results did not differ (incentive: $\beta =1.156$ [0.594, 1.719], $z=4.791$, $p<0.001$; pop-up: $\beta =1.626$ [1.087, 2.166], $z=7.024$, $p<0.001$; pop-up > incentive: $\beta =0.467$ [0.095, 0.845], $z=2.920$, $p=0.004$; see sections S2 Analyses and S6 Analyses for an in-depth exploration of participants’ search behaviour).

Did the use of lateral reading and click restraint actually improve post evaluation? And did the use of techniques mediate the effect of our interventions? To test our first question, we ran an ordinal logistic regression with accuracy score as predicted variable, and a standard logistic regression with correct guessing as predicted variable, both tests including adoption of techniques as the sole predictor. Results showed that accuracy score improved significantly if a participant reported using Civic Online Reasoning techniques ($\beta =0.526$ [0.274, 0.778], $z=4.090$, $p<0.001$). According to the model, the use of Civic Online Reasoning Techniques increased the probability of giving a “definitely valid” (“definitely invalid”) correct response by 8.8% [4.0%,14.7%]. This result however was not confirmed by the standard logistic regression on correct guessing, which instead found no significant effect of technique adoption ($\beta =0.219$ $[-0.121,0.580]$, $z=1.228$, $p=0.220$).

Based on these results, we proceeded to test whether pop-up and incentives had some mediated impact on accuracy score through technique adoption. To test mediation we used the R package MarginalMediation^70^. Technique adoption was found to mediate the effect of both incentive and pop-up on accuracy score (incentive: unstandardised $\beta =0.004$ [0.001, 0.006], $z=4.728$, $p<0.001$; pop-up: unstandardised $\beta =0.007$ [0.003, 0.012], $z=7.405$, $p<0.001$). Although testing for one mediator cannot exclude countless other explanatory variables, this analysis suggests an indirect relation between both interventions and accuracy scores.

#### Exploratory: response times

As we expected monetary incentives to increase motivation, we tested whether response times (a common proxy for increased deliberation and attention) were affected by our interventions. We compared participants’ evaluation time of the post (excluding the time spent on the pop-up) across conditions by way of a Kruskal-Wallis rank sum test. The test was significant ($\chi ^2(2)=67.63$, $p<0.001$), thus we conducted post hoc comparisons. All comparisons were significant, with participants in the incentive condition taking significantly more time than control ($\log (V)=8.02$, $p<0.001$) and pop-up ($\log (V)=5.54$, $p<0.001$) participants, and pop-up participants taking more time than control ($\log (V)=2.41$, $p=0.016$).

We tested whether longer evaluation times predicted higher accuracy scores by means of an ordinal logistic regression with log-transformed evaluation time as predictor and accuracy score as predicted variable. Results revealed a significant and positive association ($\beta =0.182$ [0.095, 0.268], $z=4.12$, $p<0.001$). The result was confirmed also for correct guessing (logistic regression, $\beta =0.242$ [0.120, 0.366], $z=3.87$, $p<0.001$).

We additionally looked at how much time participants spent outside the study page when they left without clicking any link (a proxy of lateral reading). The Kruskal-Wallis test was again significant ($\chi ^2(2)=13.482$, $p=0.001$): of those participants who performed such external searches, control participants spent less time outside the page than participants in both the incentive ($\log (V)=2.85$, $p=0.006$) and the pop-up conditions ($\log (V)=3.58$, $p=0.001$), whereas we found no significant difference between incentive and pop-up ($\log (V)=.92$, $p=0.360$).

#### Exploratory: source reputation

Civic Online Reasoning techniques were originally designed for helping to evaluate content from seemingly legitimate but unknown websites^44^. We thus analysed differences in our interventions based on the recognisability and perceived trustworthiness of the posts’ sources. The importance of a source’s perceived trustworthiness was exemplified by two posts covering the same scientific article, one from BBC News (a source trusted by most participants), and another one from the Daily Mail (a source barely trusted by most participants). Despite the posts covered the same content and presented similar wording, participants’ evaluation of the two posts differed considerably: average accuracy score was 4.7 for the BBC piece ($SD=1.05$) and 4.05 for the Daily Mail piece ($SD=1.08$; ordinal regression: $\beta =1.255$ [.926, 1.584], $z=7.470$, $p<0.001$), and the proportion of correct guesses was 90.7% and 77.3%, respectively (logistic regression: $\beta =1.059$ [0.568, 1.576], $z=4.132$, $p<0.001$).

**Figure 4:**
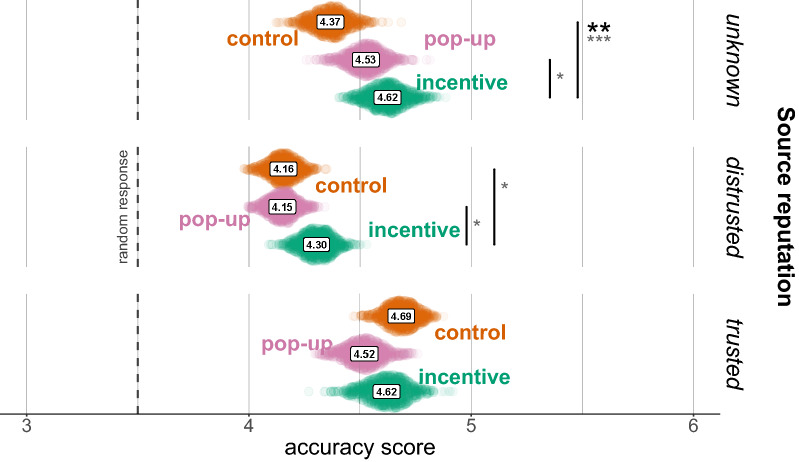
Bootstrap estimates of the average accuracy score by experimental condition and source reputation (Min. 1, Max. 6, random response: 3.5). Asterisks refer to significance of contrasts in the ordinal logistic regression. Black: family-wise corrected contrasts; dark grey: uncorrected contrasts. *$p<0.05$, **$p<0.01$, ***$p<0.001$.

Perhaps not surprisingly, we observed that, in the pop-up condition, adoption of lateral reading and click restraint was strongly linked with source type (Chi squared test with technique adoption and source category as variables, $\chi ^2(2)=15.407$, $p<0.001$): when the source was trusted, only 6.7% of participants used these techniques, whereas the proportion was 20% when the source was unknown. We then tested differences of the interventions by source type in accuracy scores and correct guessing. Likelihood-ratio tests confirmed the importance of this variable for both analyses ($p<0.001$), however family-wise corrected contrasts revealed only one significant result, the effect of incentive on accuracy scores for unknown sources ($\beta =0.558$ [0.114, 1.001], $z=3.445$, $p=0.005$; Fig. 4; see S4 Analyses for results about the uncorrected contrasts).

### Discussion

Results from Experiment 1 suggest that paying participants to be accurate does increase the accuracy score but not the proportion of participants correctly guessing the scientific validity of the posts. Exploratory analyses suggest that, compared to control, participants with an incentive gave more extreme answers, reported engaging in Civic Online Reasoning techniques more often (and did leave the page more often), spent more time in searching information outside the study page, and took longer to evaluate the post (even compared to pop-up participants). These results support the idea that monetary incentives affect accuracy, possibly by increasing motivation and attention in the task, although this hypothesis would need further testing.

By contrast, the presence of the pop-up seemed not to affect directly any indicator of accuracy. In spite of that, participants in the pop-up condition reported more lateral reading and click restraint, as well as the frequency of searches outside the study page. In turn, this increment of Civic Online Reasoning techniques (up to +13.5% when source is unknown) seems to mediate a small but significant increase in accuracy scores (exploratory marginal mediation analysis), suggesting an indirect effect of the pop-up. An effect of pop-up is possibly seen in posts produced by unknown sources, where correct guessing (but not accuracy scores) is slightly higher in the pop-up condition than in control (S4 Analyses).

These results suggest that monetary incentives might have more consistent effects over the presentation of Civic Online Reasoning techniques. At the same time, we observe considerable variability in participants’ behavior depending on specific features of the posts. For instance, source reputation seems to have a remarkable effect on the adoption of Civic Online Reasoning techniques, which were (foreseeably) overlooked by almost all participants when looking at posts from generally trusted sources.

One potential takeaway from these findings is that some prior beliefs might affect the rate at which participants look for information outside the content provided (e.g. familiarity and opinion about the source), as well as in the way they look for such information. To explore this possibility, we designed a second experiment in which we tried to reduce the influence of prior beliefs by presenting posts from generally unknown sources. Lack of source knowledge is indeed common on social media (e.g., sponsored content), and it should arguably increase the rate at which participants rely on external information. In addition, we included a fourth condition where we test the combination of monetary incentives and Civic Online Reasoning techniques, to explore whether and how the two interact.

## Experiment 2

In line with evidence in the literature, we expected an increased impact of our interventions in a context where participants could rely on less prior information. We thus conducted a second experiment that was statistically powered to test for this possibility. In the Experiment 2 we replicated the format of the first one, with two main modifications: 1) we ran a pre-screening survey to identify lesser-known sources of information and only used those sources as the basis for the Facebook posts the participants were asked to evaluate; 2) we added an experimental condition that included both incentive and pop-up interventions, to test the interaction between the two. we advanced the idea that the two intervention strategies might trigger distinct behavioral outcomes (i.e., increased time spent on the task and use of Civic Online Reasoning). If this is the case, then combining the two interventions should produce even stronger effects on accuracy. The original pre-registration of this experiment can be retrieved from osf.io/w9vfb.

### Materials and methods

#### Ethics statement

All participants gave their written informed consent for participating in the experiment. The experimental protocols were approved by the Research Ethics Committee (CER) at the University of Paris (IRB No: 00012021-05), and all research was performed in accordance with the relevant guidelines and regulations.

#### Participants

3004 U.K. residents were recruited through the online platform prolific.co on 24 May 2021 (for a rationale of sample size, see S2 Methods ). All participants gave their informed consent for participating in the experiment. Average age was 36 ($SD=13.2$, 6 not specified), 63.1% of participants were female, (36.7% male, 0.2% other), and 59.4% had a Bachelor’s degree or higher. Per our pre-registered criteria, we excluded one participant who was not a resident in the United Kingdom. Analyses were thus conducted on 3003 participants.

#### Design

The major difference from the first experiment was that sources of the Facebook posts were unknown to most participants. In addition, we included a fourth condition where we gave participants a monetary incentive and also showed them the pop-up with the Civic Online Reasoning techniques. Thus, the experiment had a between-subjects design with 2 factors, pop-up (present, absent) and monetary incentive (present, absent). Median completion time of the experiment was 5 minutes.

#### Stimuli

Participants observed one out of 6 posts that varied in terms of: the scientific validity of the content, i.e. the validity of the scientific statements in the title, subtitle, and caption of the post; the topic (climate change, coronavirus pandemic, and health and nutrition); factual reporting of the source, based on ratings from mediabiasfactcheck.com (3 high/very high versus 3 low/very low). All posts came from sources relatively unknown to participants, as measured in a preliminary survey and confirmed by participants’ familiarity ratings. There were two distinct posts for each topic, one from a source with high factual reporting displaying valid information, one from a source with low factual reporting displaying invalid information.

Some titles, subtitles and captions of the posts included references to governmental or academic institutions. To prevent that these references could affect the evaluation of the content, we slightly rephrased some sentences to remove this information. In addition, we corrected also grammatical mistakes in the text that could have given away the reliability of the source.

#### Adherence to pre-registration

We conducted our analyses as described in the original pre-registration, but some of the results for the pre-registered hypotheses are presented in the Supplementary materials. Results of pre-registered hypothesis 1 are presented in two forms, in S7 Analyses in its original formulation, and in the main text as a logistic regression (Technique adoption). Result of pre-registered hypothesis 6 is instead presented in S7 Analyses.

### Results

**Figure 5:**
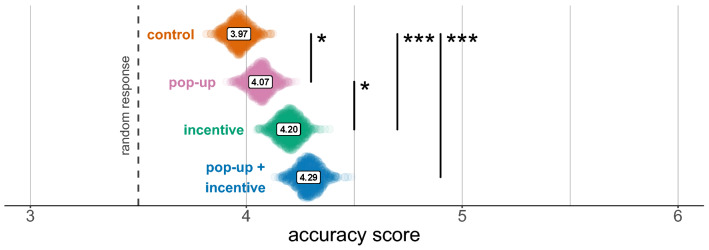
Bootstrap estimates of the average accuracy score by experimental condition (Min. 1, Max. 6, random response: 3.5). Asterisks refer to significance of contrasts in the ordinal logistic regression. *$p<0.05$, **$p<0.01$, ***$p<0.001$.

Participant randomisation was balanced across conditions (Chi squared test, $\chi ^2(1)=0.409$, $p=0.52$); average N per post, per condition was 125, minimum 106, maximum 146. Median time to evaluate the Facebook post was 33 seconds in the control condition, 48 seconds in the incentive condition, 34 seconds in the pop-up condition, and 58 seconds in the incentive + pop-up (minimum overall time: 2.5 seconds, maximum overall time: 22 minutes). When the pop-up was present, participants spent an additional median time of 11 seconds on the pop-up. On a scale from 1 to 6 (3.5 response at chance level), average accuracy score in the control condition was 3.96 ($SD=1.33$; incentive condition: 4.20, $SD=1.41$; pop-up condition: 4.07, $SD=1.33$; incentive + pop-up: 4.29, $SD=1.44$; Fig. 5). In the control condition, 64.6% of participants correctly guessed the scientific validity of the post (incentive condition: 71.2%; pop-up condition: 66.2%; incentive + pop-up: 72.9%). Overall performance was generally lower than in Experiment 1, most likely due to the use of relatively unknown news sources that forces participants not to rely on source knowledge to evaluate content.

#### Effect of interventions

To test the individual and combined effects of pop-up tips and monetary incentives (pre-registered hypothesis 3, 4, and 5) we conducted two tests, one for each accuracy index. For accuracy scores, we used two ordinal logistic regression models, one with pop-up, monetary incentive as predictors, and another regression including the same variables and the interaction between pop-up and incentive as an additional predictor. For correct guessing, we compared two logistic regressions, one with correct guessing as dependent variable and pop-up, monetary incentive as predictors, and another regression including the same variables and the interaction between pop-up and incentive as an additional predictor. For both the indices, we then adopted the model fitting data best according to a likelihood-ratio test. Perhaps surprisingly, model comparison favoured models without the interaction term (accuracy score: $\chi ^2(1)=0.032$, $p=0.858$; correct guessing: $\chi ^2(1)=0.007$, $p=.931$); we thus tested the effect of incentives and pop-up assuming that they are (approximately) orthogonal. Results revealed a significant effect of incentive on both accuracy scores ($\beta =0.350$ [0.194, 0.505], $z=5.371$, $p<0.001$) and correct guessing ($\beta =0.313$ [0.124, 0.501], $z=3.954$, $p<0.001$), and a significant effect of pop-up on accuracy scores ($\beta =0.137$ $[-0.018, 0.292]$, $z=2.115$, $p=0.034$; Mixed-effects regression with errors clustered by post: $p=0.052$), but not on correct guessing ($\beta =0.076$ $[-0.112, 0.265]$, $z=0.966$, $p=0.334$). In addition, we found that the combination of the two interventions significantly increased both accuracy indices compared to control (accuracy score: $\beta =0.487$ [0.268, 0.705], $z=5.315$, $p<0.001$; correct guessing: $\beta =0.389$ [0.123, 0.654], $z=3.496$, $p<0.001$), and that the contribution of incentive was greater than the contribution of pop-up (accuracy score: $\beta =0.213$ $[-0.007,0.432]$, $z=2.307$, $p=0.028$; correct guessing: $\beta =0.2362$ $[-0.032, 0.504]$, $z=2.103$, $p=0.047$). According to the ordinal logistic regression model, the combination of the two interventions led to a 10.4% [5.4%,14.2%] increase in correct guessing, and a 6.9% [2.8%,12.4%] increase in “definitely” correct responses compared to control.

#### Technique adoption

**Figure 6:**
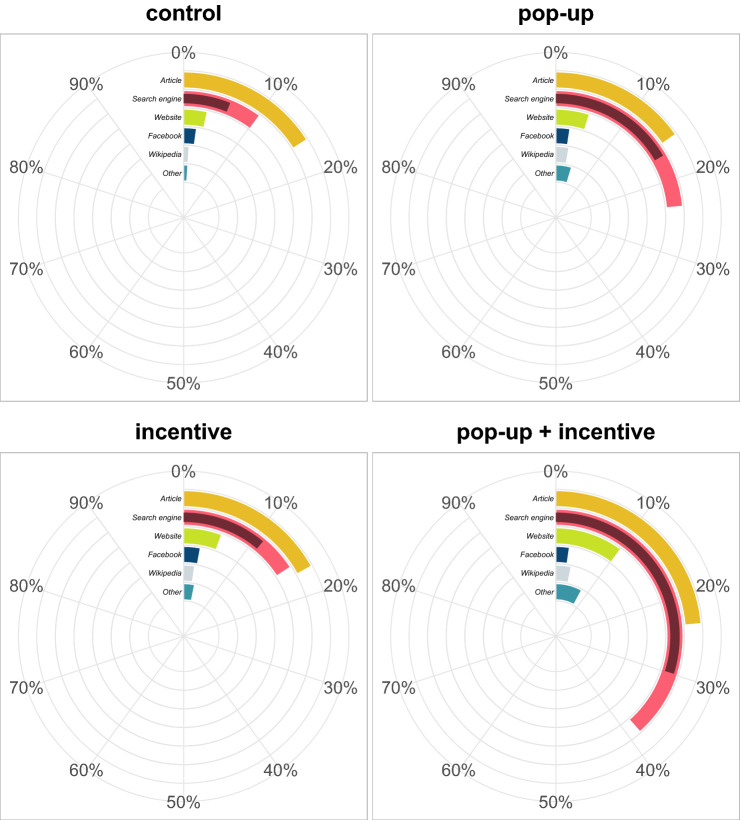
Race chart of self-report external search behaviour. Bars indicate the proportion of participants in each experimental condition reporting to have searched in either category of websites. Lateral reading is identified with the proportion of participants searching information on a search engine (light red), whereas click restraint is the subset of these participants who reported not stopping at the first algorithmically-ranked results of the search (dark red).

We tested whether technique adoption was influenced by either interventions following a similar procedure to our test for correct guessing (comparison of two logistic regressions with/without interaction; pre-registered hypothesis 1). likelihood-ratio tests again favoured the model without interaction ($\chi ^2(1)=0.245$, $p=0.621$). Model contrasts revealed several significant differences (Fig. 6): both incentive ($\beta =0.725$ [0.471, .978], $z=6.829$, $p<0.001$) and pop-up ($\beta =1.191$ [.926, 1.455], $z=10.736$, $p<0.001$) increased significantly the use of Civic Online Reasoning techniques, but pop-up effect was significantly stronger than the effect of the incentive ($\beta =0.466$ [0.106, 0.826], $z=3.093$, $p=0.002$). In addition, the combined effect of pop-up and incentive was also significant ($\beta =1.915$ [1.542, 2.288], $z=12.263$, $p<0.001$), leading to an estimated 16.5% [8.6%,26.0%] increase in technique use compared to control.

To test the robustness of these findings, we checked as in Experiment 1 the rate of false reporting (i.e., participants who said they used fact-checking techniques while they did not even leave the study page). False reporting was 22.2% in the control condition, 16% in the pop-up condition, 15.3% in the incentive condition, and 12.8% in the condition with both interventions. Exploratory analyses suggest that results did not differ after accounting for false reporting (pop-up: $\beta =1.210$ [.924, 1.496], $z=10.094$, $p<0.001$; incentive: $\beta =0.761$ [0.488, 1.033], $z=6.669$, $p<0.001$; pop-up>incentive: $\beta =0.449$ [0.061, 0.838], $z=2.759$, $p=0.006$; pop-up + incentive: $\beta =1.971$ [1.570, 2.372], $z=11.729$, $p<0.001$; see S8 Analyses for an exploration of participants’ search behaviour).

To test whether participants who adopted civic online reasoning techniques performed better in the task (pre-registered hypothesis 2) we run two tests, one for each accuracy index. For accuracy scores, since accuracy scores were non-normally distributed (Shapiro-Wilk test, all $p<0.001$) we used a ordinal logistic regression model, with accuracy score as dependent variable and adoption of techniques as a dummy predictor variable. For correct guessing, we used a logistic regression, with correct guessing as dependent variable and adoption of techniques as a dummy predictor variable. According to the models, participants adopting Civic Online Reasoning techniques were more accurate in terms of both accuracy score ($\beta =0.591$ [0.414, 0.767], $z=6.560$, $p<0.001$) and correct guessing ($\beta =0.506$ [0.281, 0.738], $z=4.345$, $p<0.001$). According to the ordinal regression model, technique adoption increased the probability of giving a “definitely valid” (“definitely invalid”) correct response increases by 9.5% [5.9%,13.7%].

We also tested whether the use of Civic Online Reasoning techniques mediated the effect of the interventions with two marginal mediation analyses on accuracy score and correct guessing. Technique adoption was found to mediate the effect of both incentive and pop-up on accuracy score (incentive: unstandardised $\beta =0.007$ [0.003, 0.010], $z=6.829$, $p<0.001$; pop-up: unstandardised $\beta =0.011$ [0.006, 0.015], $z=10.736$, $p<0.001$) and correct guessing (incentive: unstandardised $\beta =0.008$ [0.004, 0.013], $z=6.829$, $p<0.001$; pop-up: unstandardised $\beta =0.014$ [0.007, 0.021], $z=10.736$, $p<0.001$).

#### Exploratory: response times

We compared participants’ evaluation time of the post across conditions using linear regressions with rank-transformed time as dependent variable and pop-up and incentives as predictors, with and without interaction. Again, model comparison favoured the model without interaction ($F(1)=1.104$, $p=0.293$). All contrasts were significant: both incentives ($\beta =370$ [297, 443], $t(2928)=12.127$, $p<0.001$) and pop-up ($\beta =61$ $[-12,134]$, $t(2928)=2.011$, $p=0.044$) increased evaluation times, however incentives did so to a greater extent ($\beta =309$ [205, 413], $t(2928)=7.105$, $p<0.001$). Also, the combination of incentives and pop-up led to higher evaluation times than control ($\beta =431$ [329, 534], $t(2928)=10.070$, $p<0.001$). We tested whether longer evaluation times were associated with higher accuracy scores by means of an ordinal logistic regression with log-transformed evaluation time as predictor and accuracy score as predicted variable. Results revealed a significant and positive association association ($\beta =0.152$ [0.081, 0.223], $z=4.22$, $p<0.001$). The result was confirmed also for correct guessing (logistic regression, $\beta =0.204$ [0.117, 0.292], $z=4.56$, $p<0.001$). We also compared the duration of non-click external searches across conditions with the same procedure as total evaluation times, again finding no interaction between interventions ($F(1)=0.1746$, $p=0.676$). Results showed a significant effect of incentive ($\beta =52$ [15, 90], $t(726)=3.355$, $p=0.001$), pop-up ($\beta =80$ [43, 116], $t(726)=5.170$, $p<0.001$), and their combination ($\beta =132$ [80, 184], $t(726)=6.100$, $p<0.001$), but found no significant difference between the interventions ($\beta =27$ $[-26,80]$, $t(726)=1.217$, $p=0.224$).

### Discussion

Results from Experiment 2 confirmed the effectiveness of monetary incentives on accuracy, and presented evidence in favour of the potential usefulness of fact-checking tips when the post’s source is unknown. Monetary incentives increased both accuracy scores and correct guessing (pre-registered hypothesis 4), the rate of (self-reported) Civic Online Reasoning techniques, as well as the frequency and duration of non-link searches outside the study page. Participants offered with a monetary incentive spent more time evaluating the post than those who were not. Lastly, incentives seem to increase the sharing intentions of valid information compared to control (S11 Analyses ).

Contrary to the Experiment 1, the pop-up intervention seems to increase accuracy scores, but not correct guessing (pre-registered hypothesis 3). We observed that the presence of the pop-up dramatically increased technique adoption (even compared to the presence of incentives; pre-registered hypothesis 1) and the rate of non-link external searches, which in turn were linked to an increase in both measures of accuracy (pre-registered hypothesis 2). Exploratory marginal mediation analyses confirm an indirect effect of pop-up on accuracy measures via an increase of search outside the post page.

In this experiment, we also tested the interaction between incentive and pop-up (pre-registered hypothesis 5). Model comparison showed no interaction between the two interventions, suggesting that pop-up and monetary incentives contributed separately to the increase in accuracy. We additionally observe that monetary incentives increased participants’ time spent on reading the pop-up: median time is 12.3 seconds with incentive compared to 9.6 when incentive is absent (pre-registered hypothesis 6, S7 Analyses ). Despite this increase in reading times, our statistical tests do not detect an increased pop-up effects by any other metric.

## General discussion

In this research, we studied whether presenting fact-checking tips and monetary incentives increases the correct evaluation of science-themed Facebook posts. In two experiments, participants rated the scientific validity of the content of one out of several posts, with some participants receiving a monetary reward when they responded correctly and other participants being shown a pop-up window (superimposed on the Facebook post itself) that contained a list of fact-checking techniques proposed in the literature (Civic Online Reasoning). Results showed that monetary incentives work as an accuracy booster. Moreover, data on search times and extremity of validity ratings corroborated the hypothesis that incentives operate by increasing motivation and, subsequently, attention on the content and other features of the post. This effect is particularly remarkable given the strong benchmark against which it was compared: a control condition were we simply asked participants to assess the scientific validity of the content of the post. In fact, just by reading the instructions, participants in the control condition likely exerted a greater degree of attention than when routinely browsing social media^35^. The effectiveness of the pop-up as a way of introducing participants to fact-checking techniques received support in cases where the source of the post was relatively unknown, i.e. when participants could rely on low prior information to evaluate posts. Furthermore, given that the presence of the pop-up significantly increases the adoption of Civic Online Reasoning techniques, and that the use of these techniques is, in turn, a strong predictor of participants’ performance on the task, marginal mediation analyses support the hypothesis that the pop-up may have an indirect positive effect on performance.

One of the original aims of this study was to establish whether incentives and techniques could be compared in their effectiveness in improving the evaluation of scientific content, even when not directly accessible without technical expertise. In this respect, our results suggest that the presence of the pop-up has less impact on subsequent evaluation than monetary incentives. We suspect that the effectiveness of fact-checking advice may be hampered by several factors. A first explanation is that the adoption of the techniques might not have been effective enough to avoid the influence of previous beliefs about the content or of the search style. For example, if participants considered a content to be plausible in the first place, they might have selectively ignored conflicting information even when it was clearly present in the search results (i.e. confirmation bias^71^); similarly, if a participant relied primarily on certain sources of information, consulting these sources might have steered the interpretation in the wrong direction. It is unclear however how such biases might have meaningfully reduced the effectiveness of the pop-up but not of the monetary incentives. A second possibility is that participants did not engage in click restraint and instead relied only on the first few sources favoured by ranking algorithms, with the risk of not getting enough contextual information to make a correct assessment. Although we cannot be certain that more extensive searches lead to more reliable information sources, we argue that they facilitate a more balanced evaluation of the information available^72^. Lastly, the reduced impact of the pop-up may derive from its brevity: Civic Online Reasoning techniques have in fact been tested so far after being taught in extensive courses. It is therefore possible that simply presenting a condensed set of tips on the best techniques is not enough to fully understand and master them. This possibility is in line with similar unsuccessful previous interventions presenting news literacy tips^40,65,73^. Thus, true ability to recognise pseudo-scientific information might only come from a minimal mastery of critical-thinking skills, which cannot be achieved by simply adding a snippet of information to a post, in the form of a pop-up.

Despite the asymmetric contribution of monetary incentives and fact-checking techniques, our results also indicate that the interventions may work in a complementary way. In particular, Experiment 2 shows that these two interventions do not appear to interact with each other. This result, which was replicated by testing different variables of interest, suggests that the working mechanisms of the interventions are largely orthogonal, and thus can be combined to achieve an even stronger evaluation performance by participants.

Our results on incentives are in line with an attention-based account of information processing on social media; that is, increased deliberation is sufficient to decrease belief in false content^6^. Our results add to the literature of attention-based interventions by showing how monetary incentives can additionally modulate motivation and attention and increase performance.

These promising results were not self-evident, as several experiments have cautioned against the universal effectiveness of monetary incentives as a behavioural driver^74–76^. In fact, incentives that are either too small or too large have been shown to decrease rather than increase motivation^77^.

Moreover, when explicit incentives seek to modify behaviour in areas such as education, environmental actions, and the formation of healthy habits, a conflict arises between the direct extrinsic effect of incentives and how these incentives may crowd out intrinsic motivations. Seeking accuracy in judging news is certainly driven by the intrinsic motivations of individuals. In all likelihood, however, these intrinsic motivations do not conflict with monetary incentives. Seeking accuracy, unlike deliberately adopting ecological behaviour or going on a diet, is a largely automatic process.

Another concern was that motivation and attention might not have been sufficient for content that is hardly accessible to non-experts. It was thus unexpected to observe how incentives were effective even when participants evaluated information based on scientific and technical reports, and thus had to rely external knowledge and intuition when claims and data were not immediately available.

Compared to work on Civic Online Reasoning^44^, our study finds correlational and causal evidence supporting the importance of lateral reading and click restraint as predictors of accurate information, especially (as initially intended) when the information about the source is scarce. Notably, this is the first reported evidence of a general population intervention in a social media context, extending the evidence for its applicability. We note however that the connection between our intervention (the pop-up) and technique use is only indirect, as participants were free to ignore recommendations. Stronger evidence for the efficacy of Civic Online Reasoning techniques could come from within-subject studies that could limit selectively the use of the techniques to assess their direct impact on users’ behaviour.

Our results also partly support literature on media and news literacy^39^. Previous successful attempts at using fact-checking tips relied on presenting participants with some of the Facebook guidelines for evaluating information^41,42^. Critically, these tips acted by reducing post engagement (liking, commenting, sharing) and perceived accuracy of headlines by hyper-partisan and fake news sources. Given that our results highlight the effectiveness of fact-checking tips when participants are less familiar with the source, we suspect that the use of such tips is inversely associated to its knowledge and reputation: the more the source is well-known and widely respected, the less participants will rely on guidelines and recommendations. This interpretation is in line with research on media credibility cues: a site’s credentials are often seen as a sign of expertise^78^, and experimental evidence suggests that users rely on expertise^79^ and source reputation^80^ to guide their judgement. At the same time, however, studies have shown how in contexts like social media peripheral cues can be disregarded, such as clear ’sponsored content’ labels^81^. Similarly, previous studies on disinformation claim that source information has little impact on judging the accuracy of social media content^82–84^. Although we did not directly test for the presence/absence of source information, we did find that familiarity with and trust in a source largely affected the search style and evaluation of the content, suggesting that providing this information to participants had a meaningful effect on their validity evaluations. One way to reconcile these apparently antithetical conclusions is by considering the relative capability of participants to assess the plausibility of information: source knowledge can be a viable heuristic when information is harder to evaluate. Indeed, we suspect that in our experiment information about the source was often easier to assess than the plausibility of the content itself. In addition, compared to previous experiments, participants could open the original article of the post to confirm that it had actually been produced by the source and not fabricated, a factor that probably increased reliance on the source. These considerations and our findings are not sufficient to ascertain whether and under what circumstances reliance on the source is beneficial or detrimental; however, we argue that source information is important in many situations^85,86^.

Our study does not come without limitations. Possibly the most critical issue is the limited number of stimuli that were used across experiments (15), which did not allow us to properly control for many features that could impact the evaluation of the posts. Even though we cannot exclude confounding variables and biases in the selection of stimuli, we tried as much as possible to follow a standardised procedure with pre-defined criteria in order to exclude stimuli that could be considered problematic. Moreover, even though most of the literature and the present study have focused on standardised stimuli reporting content from news sources, we recognize that scientific (dis)information comes in several formats that also depend on the topic, the audience, and the strategy of the creator. We decided to exclude other types of formats (e.g. videos or screenshots) to try to minimise the differences in experience between users, we think however that future research should explore more in depth the impact of varying media on the impact of disinformation spread and on possible counteracting interventions. Another limitation to the extendibility of our results comes from the nature of the samples, consisting of UK residents recruited from an online platform, which also suggests a certain versatility with technology. Indeed, one large obstacle to the use of Civic Online Reasoning techniques is the limited or selective accessibility to Internet in several countries^87^, where navigation plans can be limited to messaging apps only. Morover, tips about lateral reading and click restraint should vary to adapt to audiences with different digital literacy, as a one-size-fit all messages have shown to be ineffective in samples with low familiarity with the online environment^41^. Future research should explore how these techniques can be proposed in contexts with limited resources, and what alternative approaches can be taken to bypass Internet constraints. Future research should explore how these techniques can be proposed in contexts with limited resources, and what alternative approaches can be taken to bypass Internet constraints. Lastly, the study explored the effectiveness of interventions when using a computer, as the very concept of lateral reading is based on browsing horizontally through internet tabs on a computer. Although nothing precludes the use of such techniques on other devices such as a mobile phone or tablet, the user interface is often not optimised to search for different contents at the same time, making their use more cumbersome. This is particularly problematic considering that social media are predominantly accessed through mobile devices. A promising direction in the fight to disinformation will be to study the influence of the device and UI in the ability of users to access high-quality information. Further studies should also investigate how much easiness of accessing information from within a specific app could prompt users to fact-check what they see. For example, many apps allow to check information on the internet via an internal browser without leaving the app itself.

## Conclusion

This study set out to assess the relative effectiveness of monetary incentives and fact-checking tips in recognising the scientific validity of social media content. We found strong evidence that incentivising participants increases accuracy evaluations; we also found evidence that fact-checking tips increase accuracy evaluation when the source of the information is unknown. These results suggest a promising role of attention and search strategies, and open the way to the test of multiple approaches in synergy to achieve the most effective results.

## Supplementary information

Supplementary Information.
