# Supplementary material for: Lateral reading and monetary incentives to spot disinformation about science
Source: Sci Rep. 2022 Apr 5;12:5678. doi: 10.1038/s41598-022-09168-y (PMC8981191; doi:10.1038/s41598-022-09168-y)
Supplement: Supplementary file 1 — Supplementary Information. [file 41598_2022_9168_MOESM1_ESM.pdf]

Supplementary Information for "Advised or Paid Way to get it right. The contribution of fact-checking tips and monetary incentives to spotting scientific disinformation"

Folco Panizza, Piero Ronzani, Simone Mattavelli, Tiffany Morisseau, Carlo Martini, Matteo Motterlini

Supporting information

S1 File. Facebook posts, Experiment 1. The spreadsheet including the list of Facebook posts for Experiment 1.

S2 File. Facebook posts, Experiment 2. The spreadsheet including the list of Facebook posts for Experiment 2.

S1 Methods. Sample size estimation, Experiment 1. Based on related findings in the literature [1], we expected a small effect size (Cohen's $d \approx .15 - .20$). Assuming no differences across the posts used as stimuli, and hence computing the sample size based on the main effect of a one-way ANOVA with three levels (experimental condition) yielded a minimum sample size of 1269 participants assuming $\alpha = 5\%$ and power $(1 - \beta) = 90\%$. Aside from the main contrast, we expected also to analyse the impact of secondary variables such as the topic of the post or trustworthiness of the source. For this reason, we planned to recruit the maximum number of participants possible given our budget constraints.

S2 Methods. Sample size estimation, Experiment 2. Our target sample size was 3000 participants. We based our sample size estimation on the main effect of pop-up on one of the two accuracy indices, correct guessing (analysis: logistic regression [2]). Estimate of this effect was based on the analyses of the first experiment (8% increase in correct guesses compared to control). To compute this effect size, we filtered observations from the first experiment based on two criteria: the source of the post had to be unknown to most participants, and participants had to have completed the task on a computer. Power $1 - \beta$ was set to 95% and significance α was set to 5%. Results yielded a sample size of $n = 733$ per condition. We thus decided to recruit 750 participants per condition, total $N = 3000$.

We further simulated achieved power for pre-registered hypotheses 3, 4 and 5, for both accuracy indices (correct guessing and accuracy score). Simulations were based on $N = 3000$, $\alpha = 5\%$, and effects sizes estimated from the first experiment.For correct guessing (test: logistic regression), achieved power is 96% for hypothesis 3 (pop-up main effect), and 88% for hypothesis 4 (incentive main effect). Combined effect of pop-up and incentive (hypothesis 5) depends on whether the two interventions interact. Therefore, we simulate different scenarios exploring the effect of interaction on power. Results reveal that to achieve at least 95% power for this contrast, the interaction effect should not be less than -4% (effect: change in the proportion of correct guesses). For accuracy scores (test: ordinal logistic regression), achieved power is 51% for hypothesis 3 (pop-up main effect), and $\approx 100\%$ for hypothesis 4 (incentive main effect). Combined effect of

pop-up and incentive (hypothesis 5) depends on whether the two interventions interact. Therefore, we simulate different scenarios exploring the effect of interaction on power. Results reveal that to achieve at least 95% power for this contrast, the interaction effect should not be less than -0.25 (effect: change in log odds).

S3 Methods. Scoring of scientific validity. Sources of scientific information usually comply with standards approved by the community to guarantee that the information provided is obtained using rigorous methods and goes through several quality checks. In order for a content to be considered scientifically valid it had to satisfy the following requirements:

- the original research could be found in a peer-reviewed publication;
- authors of the research had a track record certifying their expertise in their field of competence;
- research was not falsified by concomitant research in the field;
- there was no potential conflict of interest, or alternately the content had been independently evaluated by a source with no conflicts of interest;
- the media article represented accurately data and claims of the original research.

S4 Methods. Source familiarity and trustworthiness. Since we suspected that assessing familiarity and perceived trustworthiness of the source could be affected by the observation of the Facebook post, we ran two separate surveys with independent raters to categorise and select the Facebook posts (first survey: $N = 100$, mean age $M = 26.5$, $SD = 7.8$, 2 not specified; 71 female, 1 not specified; second survey: $N = 100$, mean age $M = 33.2$, $SD = 12.4$; 68 female, 2 not specified). Raters were recruited on the online platform prolific.co and had to assess the familiarity and trustworthiness of several sources using a questionnaire taken from a previous study [3] (Fig 1). To categorise sources based on the raters' responses, we ran an expectation maximisation model-based clustering algorithm using the McLust package in R [4]. Results revealed four clusters, one collecting known, trustworthy sources ($N = 4$; e.g., National Geographic and BBC), one known, untrustworthy sources ($N = 5$; e.g., Daily Mail and Daily Star), one unknown sources ($N = 21$; e.g., Duluth News Tribune and the American Enterprise Institute), and a last one including sources with mixed recognition ($N = 7$, e.g., the Washington Times and Live Science).

S5 Methods. Post-rating questionnaire. After rating the post's scientific validity, the participant completed a questionnaire. Below is the full list of questions asked:

- Confidence in rating: "How confident are you in your response?"; 6-point likert scale from (1) "don't know" to (6) "absolutely certain"
- Sharing intention (Experiment 2): Would you consider sharing this story online (e.g., through social networks or messaging apps)?; Yes/no
- Sharing behaviour (Experiment 2): Approximately how many news articles, memes, opinion pieces, etc. have you shared in the last week?; numeric free-text response
- Source familiarity: "Did you know [name of source] before the experiment?"; Yes/no

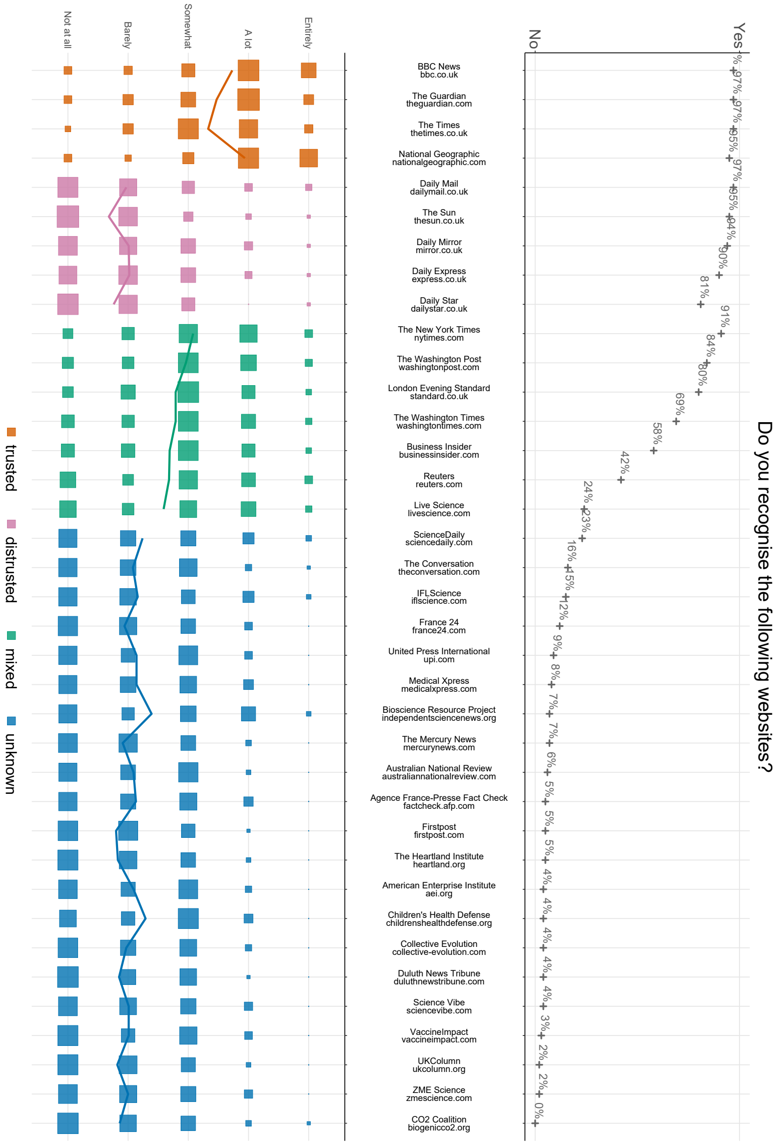

Supplementary Fig 1. Familiarity and trustworthiness ratings for a series of sources. Top: familiarity ratings, expressed as the percentage of participants recognising the source. Bottom: trust ratings, with square size indicating the proportion of participants for each response and the segmented line indicating the average rating by source. Color represents categories derived from expectation model-based clustering.

- Source trustworthiness: ”How much do you trust [name of source]?”; 5-point likert scale from (1) ”not at all” to (5) ”entirely”
- Content plausibility (Experiment 1): ”How plausible do you find the content of the post?”; 6-point likert scale from (1) ”totally implausible” to (6) ”totally plausible”
- Content plausibility (Experiment 2): ”Please respond as if you did not read the Facebook post: does it sound plausible to you that [statement based on the post]?”; 6-point likert scale from (1) ”Totally implausible” to (6) ”totally plausible”
- External search: ”While you were evaluating the Facebook post, did you look for information outside the study page?”; Yes/No
- if No: ”Why not?”; randomised: It did not occur to me to do it; I had enough information already; I thought I would lose the experiment; I thought it was not

allowed; I thought it was not possible; Other (free text entry).

- if Yes: "Where did you look for information? (select all that apply)"; randomised: The article's web page; Wikipedia; Other web pages from the article's website; Search engine (e.g., Google); Facebook. (lateral reading = search engine is selected)
- if Search engine is selected: "While you were looking at the search browser results, what links did you open?"; options: The first search results suggested.; The subsequent search results.; Both the first and subsequent search results.; I did not open any search results. (click restraint = either "The subsequent search results." or "Both the first and subsequent search results" is selected)
- Subjective knowledge of the topic [5] (study 2): "How much do you know about [topic]?"; 6-point likert scale from (1) "nothing at all" to (6) "a great deal"
- Relevance of obtaining accurate information: "We are considering compiling a comprehensive summary of the scientific discussion behind the content of the post. If so, would you be interested in receiving it by private message on your prolific account?"; Yes/No
- Trust in scientists: "In general, how much do you trust scientists to do what is right?"; 6-point likert scale from (1) "not at all" to (6) "A lot" (adapted from the Edelman Trust Barometer Yearly online survey)
- Conspiracy ideation trait [6]: 4, 5-point likert scales combined into a mean index
- Scientific literacy [5]: 15 true/false questions

S6 Methods. Supplementary measures in Experiment 2. Measures of Experiment 2 were identical to those administered in Experiment 1, with three exceptions.

Scientific validity. In Experiment 1, all the scale points used to measure scientific validity were labelled with an adjective (e.g., 4 corresponded to "possibly valid"). We removed intermediate labels and left only the ones for 1 and 6 ("definitely invalid/valid"). We removed these labels to make sure that adjectives could not influence the evaluation in the conditions with incentives, where the participants were asked to give a response that matched the ratings of the experimenters.

Plausibility. We changed one control measure, plausibility, to reflect more specifically on the content of the post than on its general appearance. We thus singled out on claim from the post and asked participants if it sounded plausible, *disregarding the information they had gathered during the task*. The content of a source should sound plausible to a participant if their background information is in agreement with the content itself, so measuring plausibility in this allows us to make inferences about a participant's background beliefs regarding the post they were given.

Sharing behaviour. As an additional exploratory measure we also asked participants' intention to share the post. This question is widely adopted in the literature (see for instance [7]). We also asked participants to estimate their weekly amount of sharing on social media, since this rate could affect the intention to share.

S1 Analyses. Original pre-registered analyses (Experiment 1). We tested differences in accuracy scores using a linear probabilistic model with accuracy score as predicted variable, and experimental condition as predictor. Contrasts revealed a small but significant impact of incentive on accuracy score ($\beta = .0264$ $[-.003, .055]$, $t(2381) = 2.133$, $p = .0495$; Mixed-effects regression with errors clustered by post:

$p = .052$), but not of the pop-up ($\beta = -.0005$ $[-.0296, .0285]$, $t(2381) = -.042$, $p = .9667$); we also found that accuracy scores were higher in the incentive condition than in the pop-up condition ($\beta = .0269$ $[-.002, .056]$, $t(2381) = 2.177$, $p = .0495$). To test correct guessing, preregistered analyses proposed the use of a probit regression. We chose however to report results of a logistic regression for ease of comparison with the other tests reported, considering that the two regressions yielded the same results. We thus used a logistic regression with the guess of participant (i.e., "valid" or "invalid") as dependent variable and actual validity of the post content, experimental condition, and their interaction as predictors. Neither experimental condition nor its interaction with post validity yielded significant results (all $p > .119$), thus we could not reject the null hypothesis that there is no difference in terms of correct guessing between conditions.

We additionally tested whether results differ when excluding participants who either failed attention checks, encountered technical issues with the display of the Facebook post, or who did not close the pop-up (and therefore could not observe the post). Tests were robust to all these exploratory exclusions.

S2 Analyses. Differences in recorded search behaviour (Experiment 1). We tracked participants' search behaviour on the post page as an additional proxy of technique use. Since the page did not include a link to a search engine, we tracked whether participants in each condition did leave the post page without clicking any link. Results confirm that more participants in the incentive and pop-up conditions left the page than participants in the control condition (Fig 2, light red bar; incentive: $\beta = .6754$ $[.3759, .9748]$, $z = 5.282$, $p < .001$; pop-up: $\beta = .5226$ $[.2182, .8270]$, $z = 4.021$, $p < .001$), however the difference between the two interventions was not significant ($\beta = -.1528$ $[-.4258, .1203]$, $z = -1.310$, $p = .190$).

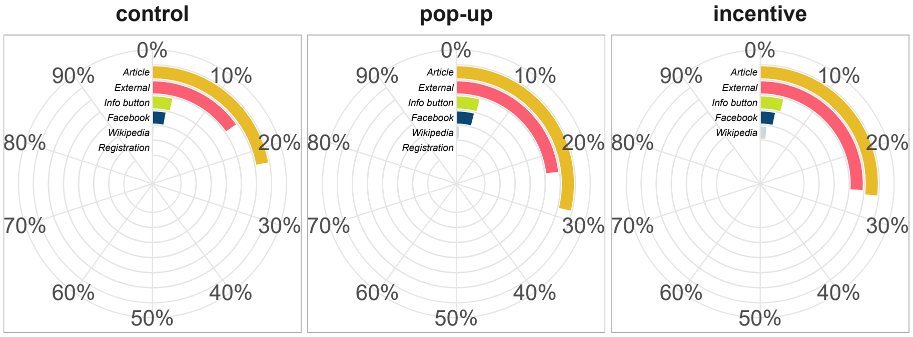

Supplementary Fig 2. Race chart of recorded search behaviour, Experiment 1. Bars indicate the proportion of participants in each experimental condition that clicked on one of the available links, on Facebook's info button, or left the page without clicking any links (named "external" searches). The links available to participants led to the original article, the source's Facebook page, a site containing the source's domain registration, and a Wikipedia page where one existed.

S3 Analyses. Response extremity and confidence (Experiment 1). We tested whether our itnerventions affected the extremity (i.e. 4 versus 5 versus 6) and confidence of ratings. To measure extremity of responses we looked at the three levels of evaluation regardless of their correctness. We ran an ordered logistic regression with extremity of response as predicted variable and experimental condition as predictor. Participants in the incentive condition gave more extreme responses than participants in the control ($\beta = .4425$ $[.2351, .6499]$, $z = 5.000$, $p < .001$) and pop-up ($\beta = .4847$ $[.27709, .6924]$, $z = 5.471$, $p < .001$) conditions, whereas we found no effect of pop-up

over control ($\beta = -.0422$ $[-.2480, .1636]$, $z = -.481$, $p = .692$). We also compared confidence ratings between conditions, but found no statistically significant difference between conditions (ordinal logistic regression, all $p > .05$).

S4 Analyses. Uncorrected contrasts for source reputation (Experiment 1). Given the smaller power for the exploratory analysis on source reputation, we looked at uncorrected contrasts. These tests suggested that, for unknown sources, accuracy scores were higher in the incentive condition than in the pop-up condition ($\beta = .3655$ $[.0843, .8153]$, $z = 2.226$, $p_{\text{uncorr}} = .026$), and that correct guessing was higher in the pop-up condition than in the control condition ($\beta = .45177$ $[-.1323, 1.0358]$, $z = 2.118$, $p_{\text{uncorr}} = .034$); for generally distrusted sources, participants in the incentive condition had higher accuracy scores than control ($\beta = .2807$ $[-.0905, .6519]$, $z = 2.071$, $p = .038$) and than pop-up participants ($\beta = .2788$ $[-.0845, .6420]$, $z = 2.102$, $p = .036$); lastly, for generally trusted sources, correct guessing was lower in the pop-up condition than in the control condition ($\beta = -.8168$ $[-1.7956, .1621]$, $z = -2.285$, $p = .022$). This last counter-intuitive result may suggest that providing Civic Online Reasoning techniques when the source is known might actually backfire. However, this interpretation should be taken with caution, since all trusted sources in the experiment were in fact presenting valid information, and thus we cannot exclude the influence of post validity (see S5 Analyses).

S5 Analyses. Effect of post type on accuracy (Experiment 1). Here we tested for any potential post differences in terms of scientific validity and scientific topic. When testing for differences across valid and invalid posts, likelihood-ratio tests confirmed the importance of this variable for accuracy scores ($chi^2(3) = 92.331$, $p < .001$) but not for correct guessing ($chi^2(3) = 5.479$, $p < .140$); we thus tested only for differences in accuracy scores. Contrasts revealed a significant effect of incentives when posts contained valid information: accuracy scores were higher in the incentive condition than in the control ($\beta = .3582$ $[.07329, .6431]$, $z = 3.268$, $p = .003$) and pop-up conditions ($\beta = .3713$ $[.0913, .6514]$, $z = 3.447$, $p = .003$). Uncorrected contrasts did not reveal any other significant result. A possible interpretation of these findings is that there was a bias in the task favouring the interpretation of the posts' content as scientifically invalid, and that the increase in time and attention produced by the incentives mitigated this bias. We do not however have the data to confirm or dis-confirm this conclusion. We also note that posts from trusted sources were all presenting valid content, and this could play a potential confound.

We then tested for differences between posts by scientific topic. Scientific topic had to have a significant effect on both accuracy scores and correct guessing (likelihood-ratio test, all $p < .001$). Contrasts reveal a significant effect of incentive on accuracy scores for posts about the COVID-19 pandemic (against control: $\beta = .4016$ $[-.0426, .8459]$, $z = 2.476$, $p = .040$; against pop-up: $\beta = .4556$ $[.0176, .8936]$, $z = 2.849$, $p = .020$) and climate change (against pop-up: $\beta = .4505$ $[.0175, .8835]$, $z = 2.850$, $p = .020$). Uncorrected contrasts did not reveal any other significant result.

S6 Analyses. Search behaviour and post evaluation (Experiment 1). As an exploratory analysis, we tested what type of behaviour on the post page predicted higher accuracy scores and correct guessing in the task. We tracked whether participants clicked on the links on the post's web page (Facebook page; original article; Facebook's info button; who.is, a website tracking information about the source domain; source's Wikipedia page, when existing), or if they left the page without clicking any links. We ran an ordinal logistic regression for accuracy score and a logistic regression for correct guessing, with predictors a series dummy variables indicating whether the

participant performed each behaviour or not. Results revealed that leaving the page without clicking any link was a significant predictor both for accuracy scores ($\beta = .4500$ $[.2649, .6352]$, $z = 4.760$, $p < .001$) and correct guessing ($\beta = .4273$ $[.1552, .7100]$, $z = 3.022$, $p = .003$). In addition, participants who opened the original article were more likely to correctly guess the validity of the post ($\beta = .4137$ $[.1600, .6754]$, $z = 3.149$, $p = .002$).

As a confirmatory test, we ran an expectation maximisation model-based clustering algorithm to categorise participants based on their tracked behaviour on the page. Specifically, we fed the algorithm with participants' total search time (either reading the info window related to the post or searching outside the page), and the proportion of time for each activity. Cluster analyses revealed four clusters of behaviours, plus a fifth group including participants who never left the study page. Results reveal that, for both accuracy scores and correct guessing, two clusters of participants performed better than those who did not leave the study page: those who predominantly searched without clicking links (accuracy score: $\beta = .5876$ $[.3932, .7820]$, $z = 5.920$, $p < .001$; correct guessing: $\beta = .6338$ $[.3493, .9317]$, $z = 4.272$, $p < .001$), and those who searched predominantly via the link to the article (accuracy score: $\beta = .3130$ $[.1203, .5056]$, $z = 3.180$, $p = .003$; correct guessing: $\beta = .4761$ $[.1969, .7671]$, $z = 3.277$, $p = .002$). We speculate (also based on comments in the post-experimental questionnaire) that participants searching on the original article used this exploration to confirm whether the post content was not fabricated, and thus rely more directly on their opinion of the source; we do not have results confirming this hypothesis.

S7 Analyses. Additional pre-registered analyses (Experiment 2).

Main effect of pop-up on adoption of techniques. To test whether the presence of the pop-up increases the adoption of civic online reasoning techniques, we used a chi squared test comparing the proportion of participants reporting to adopt the fact checking techniques (lateral reading and click restraint, dichotomous variable) when pop-up was present versus absent. Proportions were indeed significantly different ($\chi^2(1) = 122.66$, $p < .001$), with 23.6% of participants adopting lateral reading and click restraint when the pop-up was present compared to 8.7% when the pop-up was absent.

Effect of incentive on pop-up reading times. To test whether the monetary incentive increases attention towards the pop-up, we used a t-test (or an equivalent non-parametric alternative) to compare the reading times of the pop-up between participants who did or did not receive a monetary incentive. Given that reading times (and their log-transformation) were not normally distributed (Shapiro-Wilk test, all $p < .025$), we adopted a Wilcoxon rank-sum test. The test was significant ($\log(W) = 5.32$, $p < .001$), with median reading times being 2.1 [1.5,2.8] seconds longer when the incentive was present.

S8 Analyses. Differences in recorded search behaviour (Experiment 2). We checked how many participants in each condition left the post page without clicking any link, a proxy of technique use. Likelihood-ratio test again suggested no interaction between incentive and pop-up ($\chi^2(1) = .678$, $p = .410$). Results confirmed the significant effect of both incentive ($\beta = .5239$ $[.3159, .7320]$, $z = 6.010$, $p < .001$) and pop-up ($\beta = .3901$ $[.1841, .5961]$, $z = 4.519$, $p < .001$), but did not find any significant difference in strength between the two interventions ($\beta = .1339$ $[-.1579, .4256]$, $z = 1.095$, $p = .274$; Fig 3).

S9 Analyses. Exclusion criteria (Experiment 2). We tested whether results differed when excluding participants who reported being familiar with the source, or who were not regular Facebook users. Pre-registered results did not differ with one

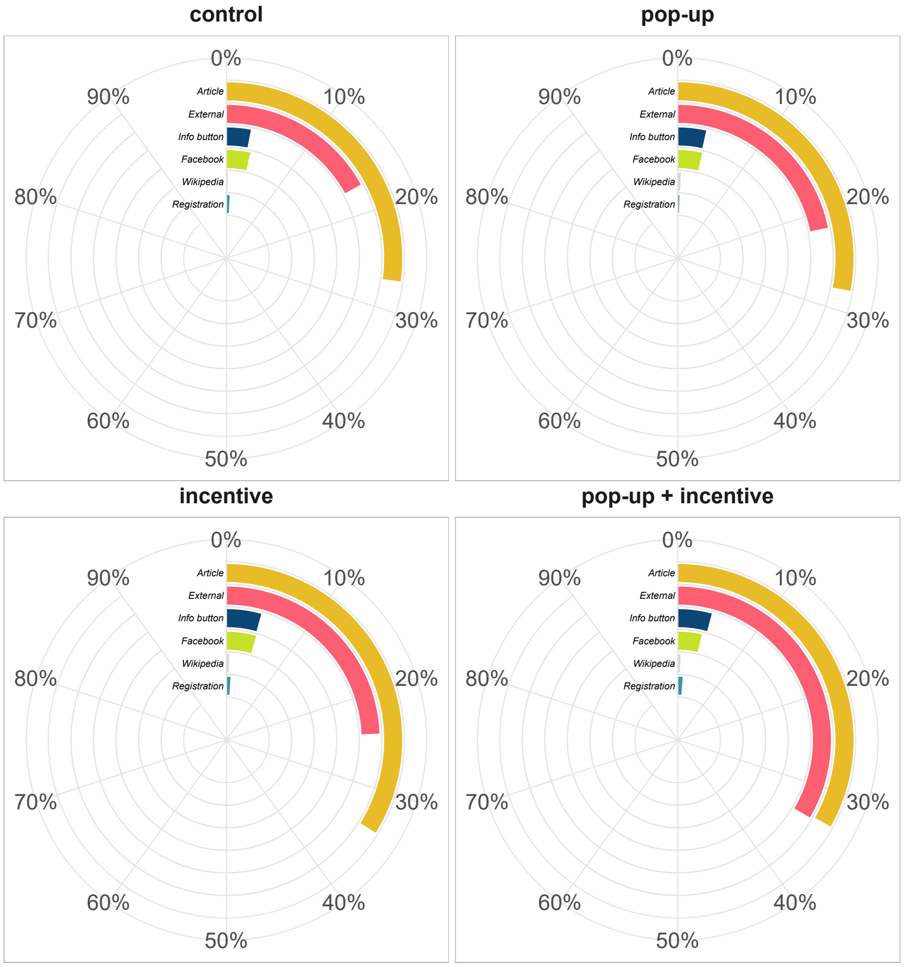

Supplementary Fig 3. Race chart of recorded search behaviour, Experiment 2.

exception: when controlling for source familiarity, the contrast comparing the strength of intervention between incentive and pop-up was no more significant (accuracy score: $\beta = .1645$ $[-.0625, .3916]$, $z = 1.730$, $p = .084$; correct guessing: $\beta = .2124$ $[-.0647, .4895]$, $z = 1.830$, $p = .090$).

S10 Analyses. Response extremity and confidence (Experiment 2). We measured differences in extremity of responses and confidence ratings across conditions as in Experiment 1. Both analyses favoured the model without interaction (likelihood-ratio tests, all $p > .05$). Contrasts for response extremity revealed that incentives increased the ratio of extreme answers ($\beta = .4963$ $[.3328, .6597]$, $z = 7.245$, $p < .001$) whereas pop-up did not ($\beta = .1191$ $[-.0434, .2815]$, $z = 1.749$, $p = .080$). Contrasts for confidence ratings revealed that only when incentive and pop-up were combined confidence ratings were significantly higher than control ($\beta = .2830$ $[.0617, .5043]$, $z = 3.053$, $p = .009$).

S11 Analyses. Sharing behaviour (Experiment 2). In Experiment 2, after the rating of the post, we asked participants about their willingness to share it. We tested the effect of incentives and pop-up on sharing behaviour. We ran two logistic regressions, one with sharing intention as predicted variable, and incentive, pop-up,

scientific validity, the interaction between incentive and scientific validity, and the interaction between pop-up and scientific validity as predictors, and a second regression identical to the first one with the additional interaction between incentive and pop-up. Both regressions included also a variable controlling for the self-report number of weekly shares of posts on social media. Given that one participant reported sharing an implausibly large number of posts (50000; S6 Methods) we excluded this participant from this analysis. Comparison of the two models favoured the model without interaction between the two interventions ($\chi^2(1) = .072$, $p = .788$). Analyses revealed only an increase in sharing intention when the post was valid and participants received a monetary incentive ($\beta = .7311$ $[.3856, 1.0765]$, $z = 5.392$, $p < .001$). One possible interpretation of this increase is that the task (assessing the scientific validity of a post) increases scepticism towards the content of the post, and that incentives counteract this scepticism by prompting people to investigate further.

Uncorrected contrasts also suggested that such increase was significantly stronger than any potential increase due to the pop-up ($\beta = .4821$ $[-.0582, 1.0224]$, $z = 2.273$, $p_{\text{uncorr}} = .023$). Moreover, pop-up appeared to slightly reduce the number of shared when the content was not valid, both compared to control ($\beta = -.3606$ $[-.7862, .0650]$, $z = -2.159$, $p_{\text{uncorr}} = .031$) and to the effect of incentive ($\beta = -.5416$ $[-1.1933, .1100]$, $z = -2.117$, $p_{\text{uncorr}} = .034$). Whereas these results indicate an influence of our interventions towards sharing behaviour, it is important to keep in mind that despite the ecological validity of this task, participants in all conditions were asked to evaluate the validity of the content which they were seeing, which could in turn influence any subsequent sharing intention [8–10].

Acknowledgments

This project has received funding from the European Union's Horizon 2020 research and innovation programme under grant agreement No 870883. The information and opinions are those of the authors and do not necessarily reflect the opinion of the European Commission. We would like to thank Torbjørn Gundersen, Philipp Lorenz-Spreen, David J. Grüning, and the members of the Prosocial Design Network for their insightful comments and advice.

References

1. Guess AM, Lerner M, Lyons B, Montgomery JM, Nyhan B, Reifler J, et al. A digital media literacy intervention increases discernment between mainstream and false news in the United States and India. Proceedings of the National Academy of Sciences. 2020;117(27):15536–15545.

2. Hsieh FY, Bloch DA, Larsen MD. A simple method of sample size calculation for linear and logistic regression. Statistics in medicine. 1998;17(14):1623–1634.

3. Pennycook G, Rand DG. Fighting misinformation on social media using crowdsourced judgments of news source quality. Proceedings of the National Academy of Sciences. 2019;116(7):2521–2526.

4. Fraley C, Raftery AE. MCLUST version 3: an R package for normal mixture modeling and model-based clustering. Washington Univ. Seattle Dept. of Satistics; 2006.

5. Fernbach PM, Light N, Scott SE, Inbar Y, Rozin P. Extreme opponents of genetically modified foods know the least but think they know the most. Nature Human Behaviour. 2019;3(3):251–256.

6. Bode L, Vraga EK. See something, say something: Correction of global health misinformation on social media. Health communication. 2018;33(9):1131–1140.

7. Pennycook G, Bear A, Collins ET, Rand DG. The implied truth effect: Attaching warnings to a subset of fake news headlines increases perceived accuracy of headlines without warnings. Management Science. 2020;.

8. Pennycook G, Binnendyk J, Newton C, Rand D. A practical guide to doing behavioural research on fake news and misinformation; 2020.

9. Pennycook G, Epstein Z, Mosleh M, Arechar AA, Eckles D, Rand DG. Shifting attention to accuracy can reduce misinformation online. Nature. 2021; p. 1–6.

10. Pennycook G, McPhetres J, Zhang Y, Lu JG, Rand DG. Fighting COVID-19 misinformation on social media: Experimental evidence for a scalable accuracy-nudge intervention. Psychological science. 2020;31(7):770–780.
